# Discordância entre Colesterol LDL e Não-HDL e Gravidade da Doença Arterial Coronária

**DOI:** 10.36660/abc.20190091

**Published:** 2020-04-06

**Authors:** Ozge Kurmus, Aycan Fahri Erkan, Berkay Ekici, Turgay Aslan, Murat Eren

**Affiliations:** 1 Ufuk University Faculty of Medicine – Cardiology Ankara Turquia Ufuk University Faculty of Medicine – Cardiology, Ankara – Turquia

**Keywords:** Doença da Artéria Coronariana,/fisiopatologia, Aterosclerose, Lipoproteinas LDL, Lipoproteinas HDL, Discordância

## Abstract

**Fundamento:**

Uma proporção considerável de pacientes apresenta níveis discordantes de colesterol de lipoproteína de baixa densidade (LDL) e de não alta densidade (não HDL).

**Objetivos:**

Avaliar a relação da discordância entre colesterol LDL e não HDL com a gravidade da doença arterial coronariana (DAC).

**Métodos:**

Avaliamos retrospectivamente os dados de 574 pacientes submetidos consecutivamente à angiografia coronariana. Foram registrados os perfis lipídicos séricos em jejum, e depois foram calculados os escores SYNTAX e Gensini para estabelecer a complexidade e a gravidade da DAC. Determinamos as medianas para colesterol LDL e não-HDL para examinar a discordância entre ambos. Discordância foi definida como LDL maior ou igual à mediana e não-HDL menor que mediana; ou LDL menor que a mediana e não-HDL maior ou igual à mediana. Valor de p < 0,05 foi aceito como estatisticamente significante.

**Resultados:**

Os níveis de colesterol LDL estiveram forte e positivamente correlacionados com os níveis de colesterol não-HDL (r = 0,865, p < 0,001), mas 15% dos pacientes apresentaram discordância entre LDL e não-HDL. A porcentagem de pacientes com escore Gensini ou SYNTAX zero não diferiu entre os grupos discordantes ou concordantes (p = 0,837, p = 0,821, respectivamente). Escores médios de Gensini e SYNTAX, porcentagem de pacientes com escore Gensini ≥ 20 e SYNTAX > 22 não foram diferentes de grupo para grupo (p = 0,635, p = 0,733, p = 0,799, p = 0,891, respectivamente). Além disso, não houve correlação estatisticamente significativa entre os escores de cholesterol LDL e Gensini ou SYNTAX em nenhum dos grupos discordantes ou concordantes. Também não foi encontrada correlação entre cholesterol não HDL e escore Gensini ou SYNTAX.

**Conclusões:**

Embora tenha havido discordância entre colesterol LDL e não-HDL (15% dos pacientes), não há diferença quanto à gravidade e complexidade da DAC entre os grupos discordantes e concordantes.

## Introdução

O colesterol de lipoproteína de baixa densidade (LDL) é um fator de risco tanto para doença cardíaca coronária de início recente como para eventos coronarianos recorrentes.1 O principal objetivo da terapia hipolipemiante é prevenir eventos ateroscleróticos.^1^^,^^2^ No entanto, apesar da obtenção de baixos níveis de colesterol LDL com tratamento ou baixos níveis basais de LDL sem tratamento, alguns pacientes ainda têm eventos adversos.^3^

A lipoproteína de não alta densidade (não HDL) contém colestrol em todas as partículas lipídicas aterogênicas potenciais, incluindo LDL, lipoproteína de densidade intermediária e lipoproteína de densidade muito baixa (VLDL). Alguns estudos sugerem que o colesterol não-HDL é um preditor mais preciso de mortalidade por doenças cardiovasculares do que o LDL.^4^^-^^6^ A recomendação é reduzir o colesterol não-HDL como meta secundária para redução de lipídios.^1^^,^^2^ Mas nem todos os pacientes têm níveis concordantes de LDL e não-HDL. Estudos demonstraram que uma proporção considerável de pacientes apresenta baixo colesterol LDL e alto não-HDL, ou alto colesterol LDL e baixo não-HDL.^7^^,^^8^

Ainda não está claro se a discordância entre os níveis de colesterol LDL e não-HDL prediz a gravidade e o prognóstico da doença arterial coronariana (DAC). Portanto, detectamos a discordância entre LDL e não-HDL e avaliamos a relação entre essa discordância e a gravidade da DAC em pacientes submetidos a angiografia coronariana.

## Métodos

### População do estudo

Este estudo retrospectivo avaliou dados de 892 pacientes submetidos a angiografia coronariana entre janeiro de 2017 e junho de 2018 em nosso laboratório de angiografia por suspeita de doença arterial coronariana estável. Dentre esses, 318 foram excluídos; 3 tinham dados incompletos, 8 apresentavam valores ausentes para qualquer medida lipídica, 6 apresentavam doença inflamatória sistêmica, insuficiência renal ou hepática, hipo/hipertireodismo ou malignidade e 301 tinham história prévia de revascularização coronariana. Por fim, incluímos os dados de 574 pacientes em nossa análise. Os parâmetros clínicos avaliados foram idade, sexo e fatores de risco coronariano. Hipertensão foi definida como pressão arterial sistólica ≥ 140 mmHg e/ou pressão arterial diastólica ≥ 90 mmHg e/ou tratamento em andamento com medicamentos anti-hipertensivos. Os pacientes foram considerados diabéticos se tivessem recebido esse diagnóstico antes do estudo e usassem medicação oral para diabetes ou fizessem tratamento com insulina no momento de admissão no estudo. O índice de massa corporal (IMC) foi calculado como peso corporal em quilogramas dividido pela altura ao quadrado em metros (kg/m^2^).

### Avaliação angiográfica

Os angiogramas diagnósticos *baseline* dos pacientes foram avaliados independentemente por dois cardiologistas intervencionistas experientes, cegos para os parâmetros lipídicos dos pacientes. O escore SYNTAX para cada paciente foi calculado através da pontuação de todas as lesões coronárias produzindo estenose ≥ 50% de diâmetro nos vasos ≥ 1,5 mm, usando o algoritmo SYNTAX, disponível no site da SYNTAX. O escore Gensini foi calculado atribuindo-se um escore de gravidade a cada estreitamento coronário com base no grau de estenose luminal e sua localização.9 Reduções no diâmetro luminal de 25%, 50%, 75%, 90%, 99% e oclusão total receberam escores 1, 2, 4, 8, 16 e 32, respectivamente.

O escore foi então multiplicado por um fator que simbolizava o significado funcional da área miocárdica suprida por esse segmento, ou seja, 5 para a artéria principal esquerda, 2,5 para a artéria descendente anterior proximal esquerda ou artéria circunflexa proximal, 1,5 para a artéria mediana anterior esquerda artéria descendente, 1 para a artéria descendente anterior distal esquerda, artéria coronária direita e artéria marginal obtusa, e 0,5 para todas as outras áreas. Em caso de desacordo em relação aos escores SYNTAX ou Gensini, um observador adicional foi consultado e a decisão final foi tomada por consenso. Escore SYNTAX baixo foi definido como ≤ 22 e escores SYNTAX intermediários e altos como > 22.^10^ Pacientes com escore Gensini ≥ 20 foram clasificados com DAC grave, equivalente a estenose de 70% ou mais na artéria descendente anterior proximal esquerda.^11^

### Avaliação laboratorial

As medições lipídicas foram realizadas em amostras de sangue coletadas dos pacientes em jejum antes da angiografia. As concentrações plasmáticas de colestrol total, LDL, HDL e triglicerídeos foram medidas por um Analisador Clínico de Bioquímica (Abbott Architect c 8000). O método colorimétrico enzimático foi utilizado para determinação quantitativa do colestrol total. O método colorimétrico terminal foi utilizado para determinação quantitativa do colesterol HDL. O colesterol LDL foi medido pelo método quantitativo colorimétrico. O método glicerol-fosfato oxidase foi utilizado para determinação quantitativa do nível de triglicerídeos. O colesterol não-HDL foi calculado subtraindo-se o nível HDL do colestrol total.

### Análise estatística

As variáveis categóricas foram expressas em números e porcentagens. A distribuição das variáveis contínuas foi considerada normal ou não com base no teste de Kolmogorov-Smirnov. Salvo indicação contrária, os dados contínuos foram descritos como média ± desvio padrão para distribuições normais e mediana (intervalo interquartil) para distribuições distorcidas. Primeiro, determinamos as medianas para colesterol LDL e não-HDL, para examinar a discordância entre elas. Categorizamos os pacientes em grupos de acordo com níveis inferiores, maiores ou iguais às medianas de colesterol LDL e não-HDL. Como não há um valor de corte padrão para discordância, escolhemos a mediana para definir a discordância e facilitar a aplicação na população estudada. Discordância foi definida como colesterol LDL maior ou igual à mediana e não-HDL menor que mediana; ou LDL menor que a mediana e não-HDL maior ou igual à mediana. Os grupos concordantes foram definidos como LDL e não-HDL maiores ou iguais à mediana, ou LDL e não-HDL menores que a mediana.

As diferenças entre as características *baseline* dos pacientes nessas categorias foram analisadas por meio do teste do qui-quadrado para comparar variáveis categóricas e o one-way ANOVA para comparar médias de medidas contínuas. O teste de Fisher *Least Significant Difference* (LSD) foi utilizado para comparações binárias. A correlação de Pearson foi usada para examinar a correlação entre variáveis contínuas, incluindo os escores de colesterol LDL, não-HDL, Gensini e SYNTAX na amostra. A correlação de Spearman foi usada para examinar as correlações desses parâmetros entre grupos concordantes e discordantes. A análise dos dados foi realizada no software SPSS for Windows, versão 22.0 (SPSS Inc., Chicago, IL, Estados Unidos). Valor de p < 0,05 foi aceito como estatisticamente significativo.

## Resultados

A idade média da população estudada foi 61,1 ± 11,4 anos e 57,5% dos 574 pacientes eram do sexo masculino. As características *baseline* são apresentadas na Tabela 1. Quase 50% dos pacientes tinham hipertensão, 30% diabetes mellitus, 32% histórico de tabagismo e um terço dos pacientes estavam em tratamento com estatina. O nível de colesterol LDL médio foi de 117,4 ± 38,3 mg/dl e o não-HDL foi 156,7 ± 46,8 mg/dl. A diferença média entre colesterol não-HDL e LDL foi de 39,2 ± 23,6 mg/dl. Pacientes com grande diferença entre os grupos não-HDL e LDL eram do sexo feminino, estavam em terapia com estatinas em menor proporção e tinham mais diabetes mellitus e altos níveis de triglicerídeos. O escore Gensini médio foi 25,3 ± 39,6, e a mediana foi 12 (0-191); o escore SYNTAX médio foi 7,1 ± 11,2 e a mediana foi 4 (0-53).

**Tabela 1:** Características baseline da população estudada

Características	
**Características clínicas**	
Sexo masculino (%)	57,5
Idade em anos (média ± desvio-padrão)	61,1 ± 11,4
Tabagismo (%)	32,1
Hipertensão (%)	49,6
Diabetes (%)	30,1
IMC (kg/m2) (média ± desvio-padrão)	28,8 ± 4,1
Uso de estatina na admissão (%)	33,3
**Análise bioquímica (média ± desvio-padrão)**	
Colesterol total (mg/dl)	198,5 ± 49,1
LDL (mg/dl)	117,4 ± 38,2
HDL (mg/dl)	41,8 ± 11,3
Triglicérides (mg/dl)	163,2 ± 84,2
Não-HDL (mg/dl)	156,7 ± 46,8
Glucose em jejum (mg/dl)	114,6 ± 40,9
Creatinina (mg/dl)	0,95 ± 0,48
**Gravidade da DAC**	
Escore Gensini médio	25,3 ± 39,6
Escore Gensini mediano (intervalo interquartil)	12 (31,1)
Escore SYNTAX médio	7,1±10,2
Escore SYNTAX mediano (intervalo interquartil)	4 (11,0)

DAC: doença arterial coronariana; LDL: colesterol de lipoproteína de baixa densidade; HDL: colesterol de lipoproteína de alta densidade; Não-HDL: colesterol de lipoproteína de densidade não-alta; IMC: índice de massa corporal.

**Figura 1:**
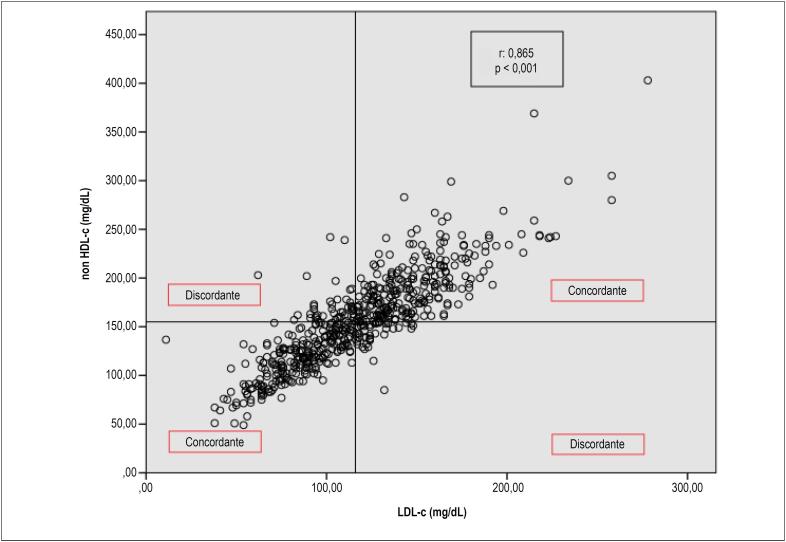
Gráficos de dispersão e prevalência de discordância e concordância definidas de acordo com os valores medianos de colesterol LDL e não-HDL. LDL: colesterol de lipoproteína de baixa densidade; Não-HDL: colesterol de lipoproteína de alta densidade.

Os níveis de LDL estiveram forte e positivamente correlacionados com os níveis de não-HDL (r = 0,865, p < 0,001), mas houve discordância entre ambos. Essa discordância foi encontrada em 15% dos pacientes. A magnitude da discordância e distribuição dos níveis de colesterol LDL e não-HDL de acordo com as medianas são mostradas na Figura 1. O colesterol não-HDL foi correlacionado com o triglicerídeo (TG) (r = 0,431, p < 0,001). O escore Gensini foi fortemente correlacionado com o escore SYNTAX (r = 0,927, p < 0,001). Porém, nem o escore Gensini nem o SYNTAX foram correlacionados com colesterol LDL (p = 0,9 e p = 0,9, respectivamente). Os escores também não foram correlacionados com o colesterol não-HDL (p = 0,4 e p = 0,4, respectivamente).

Para avaliar melhor as características dos pacientes com discordância e concordância entre colesterol LDL e não-HDL, classificamos os pacientes em 4 subgrupos. Grupo 1: LDL < mediana e não-HDL < mediana, grupo 2: LDL < mediana e não-HDL ≥ mediana, grupo 3: LDL ≥ mediana e não-HDL < mediana, grupo 4: LDL ≥ mediana e não-HDL ≥mediana. Os grupos 2 e 3 foram grupos discordantes (Tabela 2).

**Tabela 2:** Características dos pacientes com colesterol LDL e não-HDL concordante e discordante

	LDL < mediana não-HDL < mediana n = 245 (grupo 1)	LDL < mediana não-HDL ≥ mediana n = 43 (grupo 2)	LDL ≥ mediana não-HDL < mediana n = 43 (grupo 3)	LDL ≥ mediana não-HDL ≥ mediana n = 243 (grupo 4)	Valor de p
Idade (anos)	62,0 ± 12,5	58,6 ± 11,7	61,4 ± 10,8	60,7 ± 10,2	0,266
Sexo feminino (%)	35,9	41,9	44,2	49,0	0,036
Tabagismo (%)	34,3	30,2	30,2	30,6	0,818
Hiperensão (%)	50,6	53,5	41,9	49,2	0,704
Diabetes (%)	34,7	46,5	20,9	24,3	0,004
Tratamento com estatina (%)	45,3	18,6	30,2	24,4	0,001
IMC (kg/m2)	28,5 ± 4,0	29,1 ± 4,9	29,1 ± 3,0	29,0 ± 4,2	0,501
Colesterol total (mg/dl)	156,4 ± 27,2	208,2 ± 20,4	190,1 ± 16,8	240,7 ± 35,3	< 0,001^a,b,c,d,e,f^
LDL (mg/dl)	84,2 ± 18,9	103,0 ± 11,3	126,6 ± 8,5	151,8 ± 26,8	< 0,001^a,b,c,d,e,f^
HDL (mg/dl)	40,1 ± 11,7	36,1 ± 9,5	46,6 ± 13,4	43,7 ± 10,1	< 0,001^a,b,c,d,e,f^
Não-HDL (mg/dl)	116,4 ± 23,4	172,1 ± 19,2	143,4 ± 13,1	197,0 ± 34,6	< 0,001^a,b,c,d,e,f^
Triglicérides (mg/dl)	132,0 ± 81,6	256,1 ± 118,3	127,8 ± 60,4	184,5 ± 96,5	< 0,001^a,,c,d,e,f^
Glicose em jejum (mg/dl)	121,1 ± 50,4	119,4 ± 40,4	107,4 ± 20,9	108,5 ± 30,9	0,003 ^b,c^
Escore Gensini médio	24,7 ± 38,1	28,2 ± 36,4	18,7 ± 28,1	26,5 ± 40,1	0,635
Escore SYNTAX médio	7,1 ± 11,2	6,7 ± 11,3	5,4 ± 9,3	7,4 ± 11,6	0,733
Escore Gensini = 0 (%)	24,9	30,2	23,3	23,9	0,837
Escore SYNTAX = 0 (%)	55,1	60,5	58,1	54,3	0,821
Escore Gensini ≥ 20 (%)	34,7	27,9	30,2	34,2	0,799
Escore SYNTAX > 22 (%)	13,5	9,3	11,6	12,8	0,891

Dados expressos em porcentagem para variáveis categóricas; teste qui-quadrado foi utilizado. Dados expressos em média±desvio-padrão para variáveis contínuas; one-way ANOVA; Valores de p estatisticamente significativos estão em negrito. O teste LSD foi realizado para comparações binárias entre os grupos e o valor de p foi fixado em 0,05. Foram encontradas diferenças significativas entre a) grupo I e grupo II, b) grupo I e grupo III, c) grupo I e grupo IV, d) grupo II e grupo III, e) grupo II e grupo IV, f) grupo III e grupo IV. LDL: colesterol de lipoproteína de baixa densidade; HDL: colesterol de lipoproteína de alta densidade; Não-HDL: colesterol de lipoproteína de densidade não-alta; IMC: índice de massa corporal.

As variáveis idade, IMC, histórico de tabagismo e porcentagem de pacientes com hipertensão não foram diferentes entre os grupos. As porcentagens de pacientes com diabetes mellitus e em tratamento com estatina foram significativamente diferentes entre os grupos (p = 0,004 e p < 0,001, respectivamente). O grupo 2 (LDL < mediana e não-HDL ≥ mediana) teve a maior prevalência de diabetes mellitus e a menor de tratamento em andamento com estatina. A porcentagem mais alta de pacientes em tratamento com estatina foi do grupo 1 (LDL < mediana e não-HDL < mediana).

O sexo foi significativamente diferente de um grupo a outro (p = 0,036). O grupo 1 teve a menor porcentagem de mulheres (LDL < mediana e não-HDL < mediana), enquanto o grupo 4 teve a maior (LDL ≥mediana e não-HDL ≥ mediana). O colesterol total e o LDL estiveram presentes em altas proporções nos grupos com LDL ≥ mediana e não-HDL ≥ mediana, mas os níveis de triglicerídeos foram os mais altos no grupo com LDL < mediana e não-HDL ≥ mediana (p < 0,001, p < 0,001 e p < 0,001, respectivamente).

A porcentagem de pacientes com escore Gensini ou SYNTAX igual a zero não diferiu entre os grupos (p = 0,837 e p = 0,821, respectivamente). Os escores Gensini e SYNTAX médios, a porcentagem de pacientes com escore Gensini ≥ 20 e SYNTAX > 22 também não diferiram entre grupos (p = 0,635, p = 0,733, p = 0,799 e p = 0,891, respectivamente). Também não houve correlação estatisticamente significativa entre o colesterol LDL e o escore Gensini ou SYNTAX em nenhum dos quatro subgrupos. Também não foi encontrada correlação entre colesterol não-HDL e os escores Gensini ou SYNTAX nos subgrupos (Tabela 3).

**Tabela 3:** Correlação dos níveis de LDL, não-HDL, escores Gensini e SYNTAX com rho de Spearman e valor de p

	LDL < median Não-HDL < mediana n = 245 (grupo 1)		LDL < median Não-HDL ≥ mediana n = 43 (grupo 2)		LDL ≥ median Não-HDL < mediana n = 43 (grupo 3)		LDL ≥ median Não-HDL ≥ mediana n = 243 (grupo 4)	
	Escore Gensini	Escore SYNTAX	Escore Gensini	Escore SYNTAX	Escore Gensini	Escore SYNTAX	Escore Gensini	Escore SYNTAX
LDL	r = 0,118	r = 0,101	r = 0,088	r = 0,18	r = 0,127	r = 0,029	r = 0,031	r = 0,002
	p = 0,064	p = 0,115	p = 0,577	p = 0,910	p = 0,418	p = 0,853	p = 0,635	p = 0,972
Não-HDL	r = 0,046	r = 0,031	r = 0,190	r = 0,165	r = 0,104	r = 0,183	r = 0,025	r = 0,034
	p = 0,469	p = 0,624	p = 0,221	p = 0,290	p = 0,506	p = 0,240	p = 0,694	p = 0,596

LDL: colesterol de lipoproteína de baixa densidade; HDL: colesterol de lipoproteína de alta densidade; Não-HDL: colesterol de lipoproteína de densidade não-alta.

## Discussão

No presente estudo, avaliamos a associação transversal entre gravidade/complexidade da DAC e discordância entre os níveis de colesterol LDL e não-HDL. Embora houvesse discordância entre colesterol LDL e não-HDL em pacientes submetidos a angiografia coronariana (15% da amostra), não houve diferença quanto à gravidade e complexidade da DAC entre os grupos discordantes e concordantes.

O colesterol não-HDL representa o conteúdo de colesterol de todas as lipoproteínas aterogênicas circulantes e não é influenciado pelas condições de jejum. Vários estudos indicaram que se trata de melhor preditor de risco cardiovascular e mortalidade que o LDL.^4^^,^^5^^,^^12^^,^^13^ Também foi relatado que o colesterol não-HDL esteve mais associado a eventos cardiovasculares do que o LDL em pacientes utilizando estatina.^3^^,^^14^ Existem algumas explicações para esses achados. Em primeiro lugar, o colesterol não-HDL inclui colesterol VLDL e LDL, e o VLDL também é aterogênico.^15^^,^^16^ Em segundo lugar, o não-HDL é uma medida indireta das partículas de LDL (LDL-p), e o risco aterosclerótico relacionado ao LDL é mais bem determinado pelo nível de LDL-p.^17^^-^^19^ Finalmente, o não-HDL está correlacionado com a apolipoproteína B (ApoB).^20^ As lipoproteínas portadoras de ApoB iniciam e mantêm o processo aterosclerótico dentro da parede arterial, de modo que o número total de partículas de ApoB é um determinante crítico do risco cardiovascular.^5^^,^^21^^-^^23^ Para calcular o colesterol não HDL, nenhuma medida adicional além dos parâmetros lipídicos de rotina é necessária; portanto, não há despesas adicionais, uma vantagem do colesterol não HDL em relação à ApoB.

O LDL-p pode ser empobrecido ou enriquecido com colesterol. Essa variação causa discordância entre LDL e não-HDL. A taxa de discordância em nosso estudo é semelhante à de estudos anteriores. Em um estudo com 27.533 participantes, houve 11,6% de discordância e, em outro com 1.757 pacientes, 14,6%.^7^^,^^8^ Também em estudo realizado com aproximadamente 1,3 milhão de adultos, a taxa de discordância foi semelhante (15%), principalmente com níveis mais baixos de LDL.^24^ A discordância éalta entre indivíduos com alto nível de triglicerídeos, HDL mais baixo, disglicemia e obesidade.^7^^,^^25^^,^^26^

O risco coronariano foi subestimado ou superestimado pelo colesterol LDL em indivíduos com discordância.7 Tanto o LDL quanto o não-HDL e a discordância com eventos cardiovasculares futuros foram avaliados em vários estudos. No entanto, dados sobre parâmetros lipídicos ou discordância que predizem com precisão a gravidade ou complexidade da aterosclerose coronariana são limitados e controversos.

Em estudo realizado por Budde et al.,^27^ não houve relação entre colesterol LDL e número, gravidade e extensão das lesões coronárias.^27^ Além disso, não houve relação entre o LDL e o volume da placa coronariana, doença coronariana principal de 3 vasos ou esquerda e estenose coronariana grave.^28^ No estudo de Onat et al.,^29^ O colesterol LDL não foi preditor de doença cardíaca coronária de início recente.^29^ Em dois estudos que avaliaram a relação entre escore Gensini e LDL, o LDL-C não teve diferença significativa quando comparado aos escores alto e baixo de Gensini.^30^^,^^31^ Em nosso estudo, o nível de LDL não se correlacionou com os escores Gensini ou SYNTAX. Verificou-se que o não-HDL não era maior em pacientes com escore Gensini igual ou superior a 50 em comparação a pacientes com escore Gensini inferior a 50.^30^ Houve uma fraca correlação (r = 0,113, p < 0,001) entre o não-HDL e o escore Gensini no estudo de Zhang e et al.^8^ Em nosso estudo, foi pequena a proporção de pacientes com altos escores SYNTAX e Gensini. A falta de associação entre gravidade da DAC e colesterol não-HDL pode ter resultado do número relativamente limitado de pacientes com DAC grave na amostra.

Há um número limitado de estudos que avaliam o efeito da discordância entre colesterol LDL e não-HDL na gravidade da aterosclerose coronariana. Verificou-se que o escore de Gensini foi superestimado entre os pacientes com LDL mais alto ou igual à mediana e não-HDL abaixo da mediana.^8^ Shiiba et al.,^32^ avaliaram a relação entre essa discordância e o resultado a médio prazo de implante de *stent* coronário. Verificou-se que a doença de três vasos ou doença do trato principal esquerdo não diferiu entre os grupos discordantes e concordantes, e a discordância entre os níveis de LDL e não-HDL não foi preditora de grandes eventos cardiovasculares adversos após o implante de *sten.t*^32^ Avaliamos a gravidade da DAC por meio do escore Gensini e a complexidade pelo escore SYNTAX, e estes não diferiram entre os grupos discordantes e concordantes em nosso estudo.

### Limitações do estudo

Este estudo tem várias limitações. Por exemplo, seu desenho retrospectivo abre caminho para a possibilidade de viés de fatores cofundadores não mensurados. Um terço dos pacientes estava em tratamento com estatinas e a falta de associação entre discordância e gravidade da DAC pode ter sido decorrente disso. Além disso, faltavam informações sobre doses, tipo e duração do tratamento com estatinas. Não há definição absoluta e valores de corte padrão para a discordância de colesterol LDL e não-HDL. Utilizamos valores medianos para a população estudada. Portanto, mais estudos prospectivos em larga escala seriam necessários para validar nossos resultados.

## Conclusão

Houve discordância entre colesterol LDL e não-HDL (15% dos pacientes), porém não há diferença quanto à gravidade e complexidade da DAC entre os grupos discordantes e concordantes. No entanto, os pacientes com colesterol LDL < mediana e não-HDL ≥ mediana apresentam algumas características de alto risco, como diabetes mellitus e níveis mais altos de triglicerídeos, podendo necessitar de mais avaliações e um acompanhamento estrito.
