# Supplementary material for: Discordância entre Colesterol LDL e Não-HDL e Gravidade da Doença Arterial Coronária
Source: Arq Bras Cardiol. 2020 Apr 6;114(3):469–75. [Article in Portuguese] doi: 10.36660/abc.20190091 (PMC7792727; doi:10.36660/abc.20190091)
Supplement: Translation

# Translation: Discordance of Low-Density Lipoprotein Cholestrol and Non-High-Density Lipoprotein Cholestrol and Coronary Artery Disease Severity

**Authors:** Ozge Kurmus, Aycan Fahri Erkan, Berkay Ekici, Turgay Aslan, Murat Eren

**Affiliations:** 1 Ankara Turkey Ufuk University Faculty of Medicine – Cardiology, Ankara – Turkey

**Keywords:** Coronary Artery Disease/physiopathology, Atherosclerosis, Lipoproteins, LDL, Lipoproteins, HDL, Discordance

## Abstract

**Background:**

A sizeable proportion of patients have discordant low-density lipoprotein cholesterol (LDL-C) and non-high-density lipoprotein cholesterol (non-HDL-C).

**Objectives:**

We assessed the relationship between discordance of LDL-C and non-HDL-C and coronary artery disease (CAD) severity.

**Methods:**

We retrospectively evaluated the data of 574 consecutive patients who underwent coronary angiography. Fasting serum lipid profiles were recorded, SYNTAX and Gensini scores were calculated to establish CAD complexity and severity. We determined the medians for LDL-C and non-HDL-C to examine the discordance between LDL-C and non-HDL-C. Discordance was defined as LDL-C greater than or equal to the median and non-HDL-C less than median; or LDL-C less than median and non-HDL-C greater than or equal to median. A p value < 0.05 was accepted as statistically significant.

**Results:**

LDL-C levels were strongly and positively correlated with non-HDL-C levels (r = 0.865, p < 0.001) but 15% of patients had discordance between LDL-C and non-HDL-C. The percentage of patients with a Gensini score of zero or SYNTAX score of zero did not differ between discordant or concordant groups (p = 0.837, p = 0.821, respectively). Mean Gensini and SYNTAX scores, percentage of patients with Gensini score ≥20 and SYNTAX score >22 were not different from group to group (p = 0.635, p = 0.733, p = 0.799, p = 0.891, respectively). Also, there was no statistically significant correlation between LDL-C and Gensini or SYNTAX scores in any of the discordant or concordant groups. Additionally, no correlation was found between non-HDL-C and Gensini or SYNTAX score.

**Conclusions:**

While there was discordance between LDL-C and non-HDL-C (15% of patients), there is no difference regarding CAD severity and complexity between discordant and concordant groups.

## Introduction

Low-density lipoprotein-cholestrol (LDL-C) is a risk factor for both new-onset coronary heart disease and recurrent coronary events.^1^ The main target of lipid-lowering therapy is to prevent atherosclerotic events.^1^^,^^2^ However, despite the achivement of low levels of LDL-C with treatment or having basal low levels of LDL without treatment, some patients still experience adverse events.^3^

Non-high-density lipoprotein cholestrol (non-HDL-C) contains cholestrol in all potential atherogenic lipid particles including LDL, intermediate-density lipoprotein and very-low-density lipoprotein (VLDL). Some studies suggest that non-HDL-C is a better predictor of cardiovascular disease mortality than LDL-C.^4^^-^^6^ The recommendation is to reduce non-HDL-C as a secondary lipid-lowering target.^1^^,^^2^ But not all patients have concordant LDL-C and non-HDL-C levels. Studies have shown that a sizeable proportion of patients presents low LDL-C and high non-HDL-C or high LDL-C and low non-HDL-C.^7^^,^^8^

It is not yet clear whether the discordance between LDL-C and non-HDL-C predicts severity and prognosis of coronary artery disease (CAD). Therefore, we detected the discordance of LDL-C and non-HDL-C, and assessed the relationship between this discordance and CAD severity in patients who had undergone coronary angiography.

## Methods

### Study Population

This retrospective study assessed the data of 892 patients who had undergone coronary angiography between January 2017 and June 2018 in our angiography laboratory because of suspected stable coronary artery disease. Among these, 318 patients were excluded; 3 had incomplete data, 8 had missing values for any lipid measurements, 6 had systemic inflammatory disease, renal or hepatic failure, hypo/ hyperthyrodism or malignancy, and 301 had previous history of coronary revascularization. Finally, we included the data of 574 patients in our analysis. The clinical parameters assessed were age, gender and coronary risk factors. Hypertension was defined as systolic blood pressure ≥ 140 mmHg and/or diastolic blood pressure ≥ 90 mmHg and/or current medication with antihypertensive drugs. Patients were defined as diabetic if they had been informed of this diagnosis prior to the study and had been using oral antidiabetic drugs or insulin treatment upon study admission. Body mass index (BMI) was calculated as body weight in kilograms divided by the squared height in meters (kg/m^2^).

### Angiographic Evaluation

Baseline diagnostic angiograms of the patients were assessed independently by two experienced interventional cardiologists who were blinded to patients' lipid paremeters. SYNTAX score for each patient was calculated by scoring all coronary lesions producing ≥ 50% diameter stenosis in vessels ≥ 1.5 mm using the SYNTAX score algorithm, which was available on the SYNTAX website. Gensini score was calculated by assigning a severity score to each coronary narrowing on the basis of the degree of luminal stenosis and its location.^9^ Decreases in luminal diameter of 25%, 50%, 75%, 90%, 99%, and total occlusion were given scores of 1, 2, 4, 8, 16 and 32, respectively. The score was then multiplied by a factor symbolizing the functional significance of the myocardial area supplied by that segment, that is, 5 for the left main artery, 2.5 for the proximal left anterior descending artery or proximal circumflex artery, 1.5 for the mid left anterior descending artery, 1 for the distal left anterior descending artery, right coronary artery and obtuse marginal artery, and 0.5 for all other areas. In cases of disagreement regarding SYNTAX or Gensini scores, an additional observer was consulted and the final decision was made by consensus. A low SYNTAX score was defined as ≤ 22, while intermediate and high SYNTAX scores were set as > 22. ^10^ Patients with Gensini score of ≥20 were defined as severe CAD, which was approximately equal to having a 70% or more stenosis in the proximal left anterior descending artery.^11^

### Laboratory Measurements

Lipid measurements were performed on fasting blood samples taken before the angiography. Plasma concentrations of total cholestrol, LDL-C, HDL-C and triglycerides were measured with a Clinical Biochemistry Analyzer (Abbott Architect c 8000). The enzymatic colorimetric method was used for quantitative determination of total cholestrol. The endpoint colorimetric method was used for quantitative determination of HDL-C. LDL-C was measured by quantitative colorimetric method. The glycerol phosphate oxidase method was used for quantitative determination of triglycerides level. Non-HDL-C was calculated as total cholestrol minus HDL-C.

### Statistical analysis

Categorical variables were defined as numbers and percentages. The distribution of continuous variables was considered as normal or not based on the Kolmogorov-Smirnov test. Unless specified otherwise, continuous data were described as mean ± standard deviation for normal distributions, and median (interquartile range) for skewed distributions. First, we determined the medians for LDL-C and non-HDL-C to examine the discordance between them. We categorized patients into groups according to less than, greater than or equal to median levels of LDL-C and non-HDL-C. As there is no standard cutoff point for discordance, we chose the median to define discordance and to make it easier to apply to our study population. Discordance was defined as LDL-C greater than or equal to the median, and non-HDL-C as less than median; or LDL-C less than median and non-HDL-C greater than or equal to median. Concordant groups were defined as both LDL-C and non-HDL-C greater than or equal to median, or both LDL-C and non-HDL-C less than median. Differences between baseline characteristics of patients across these categories were analyzed with the chi-square test for comparing categorical variables and the One-way ANOVA for comparing means of continuous measures. The LSD test was used for binary comparisons. Pearson's correlation analysis was used to examine the correlation between continuous variables, including LDL-C, non-HDL-C, Gensini and SYNTAX scores in the sample. Spearman's correlation was used to examine correlations between these parameters in concordant and discordant groups. Data analyses were performed on SPSS for Windows, version 22.0 (SPSS Inc., Chicago, IL, United States). A p value < 0.05 was accepted as statistically significant.

## Results

The mean age of the study population was 61.1 ± 11.4 years, and 57.5% of the 574 patients were males. Baseline characteristics are presented in Table 1. Nearly 50% of the patients had hypertension, 30% had diabetes mellitus, 32% had past smoking history, and one third of the patients were on statin treatment. Mean LDL-C was 117.4 ± 38.3 mg/dl and non-HDL-C was 156.7 ± 46.8 mg/dl. Mean difference between non-HDL-C and LDL-C was 39.2±23.6 mg/dl. Patients with high difference between non-HDL-C and LDL-C were more commonly females, receiving less statin therapy and having more diabetes mellitus and high triglycerides levels. Mean Gensini score was 25.3 ± 39.6, and the median was 12 (0-191); mean SYNTAX score was 7.1 ± 11.2, and the median was 4 (0-53).

**Table 1:** Baseline characteristics of the study population

Characteristics	
**Clinical characteristics**	
Male gender (%)	57.5
Age in years (mean ± standart deviation)	61.1 ± 11.4
Smoking (%)	32.1
Hypertension (%)	49.6
Diabetes (%)	30.1
BMI (kg/m2) (mean ± standart deviation)	28.8 ± 4.1
Statin usage at admission (%)	33.3
**Biochemical analysis (mean ± standart deviation)**	
Total cholesterol (mg/dl)	198.5 ± 49.1
LDL-C (mg/dl)	117.4 ± 38.2
HDL-C (mg/dl)	41.8 ± 11.3
Triglyceride (mg/dl)	163.2 ± 84.2
Non-HDL-C (mg/dl)	156.7 ± 46.8
Fasting glucose (mg/dl)	114.6 ± 40.9
Creatinine (mg/dl)	0.95 ± 0.48
**CAD severity**	
Mean Gensini score	25.3 ± 39.6
Median Gensini score (interquartile range)	12 (31.1)
Mean SYNTAX score	7.1±10.2
Median SYNTAX score (interquartile range)	4 (11.0)

CAD: coronary artery disease; LDL-C: low-density lipoprotein cholesterol; HDL-C: high-density lipoprotein cholesterol; Non-HDL-C: non-high-density lipoprotein cholesterol; BMI: body mass index.

LDL-C levels were strongly and positively correlated with non-HDL-C levels (r=0.865, p < 0.001), but there was discordance between them. Discordance of LDL and non-HDL-C was found in 15% of patients. The magnitude of discordance and distribution of LDL-C and non-HDL-C levels according to medians are shown in Figure 1. Non-HDL-C was correlated with triglyceride (TG) (r = 0.431, p < 0.001). Gensini score was highly correlated with SYNTAX score (r = 0.927, p < 0.001). Neither Gensini nor SYNTAX were correlated with LDL-C (p = 0.9 and p = 0.9, respectively). Also, both scores were not correlated with non-HDL-C (p = 0.4 and p = 0.4, respectively).

**Figure 1:** Scatterplots and prevalence of discordance and concordance defined according to median values of LDL-C and non-HDL-C. LDL-C: low-density lipoprotein cholesterol; Non-HDL-C: non-high-density lipoprotein cholesterol.

To further evaluate the characteristics of patients with discordance and concordance of LDL-C and non-HDL-C, we classified patients into 4 subgroups. Group 1: LDL-C < median and non-HDL-C <median, group 2: LDL-C <median and non-HDL-C ≥ median, group 3: LDL-C ≥median and non-HDL-C < median, group 4: LDL-C ≥ median and non-HDL-C ≥ median. Groups 2 and 3 were discordant groups (Table 2).

**Table 2:** Characteristics of patients with concordant and discordant LDL-C and non-HDL-C

	LDL-C < median non-HDL-C < median n = 245 (group 1)	LDL-C < median non-HDL-C ≥ median n = 43 (group 2)	LDL-C ≥ median Non-HDL-C < median n = 43 (group 3)	LDL-C ≥ median Non-HDL-C ≥ median n = 243 (group 4)	p-value
Age (years)	62.0 ± 12.5	58.6 ± 11.7	61.4 ± 10.8	60.7 ± 10.2	0.266
Female gender (%)	35.9	41.9	44.2	49.0	0.036
Smoking (%)	34.3	30.2	30.2	30.6	0.818
Hypertension (%)	50.6	53.5	41.9	49.2	0.704
Diabetes (%)	34.7	46.5	20.9	24.3	0.004
Receiving statin (%)	45.3	18.6	30.2	24.4	0.001
BMI (kg/m2)	28.5 ± 4.0	29.1 ± 4.9	29.1 ± 3.0	29.0 ± 4.2	0.501
Total cholesterol (mg/dl)	156.4 ± 27.2	208.2 ± 20.4	190.1 ± 16.8	240.7 ± 35.3	< 0.001^a,b,c,d,e,f^
LDL-C (mg/dl)	84.2 ± 18.9	103.0 ± 11.3	126.6 ± 8.5	151.8 ± 26.8	< 0.001^a,b,c,d,e,f^
HDL-C (mg/dl)	40.1 ± 11.7	36.1 ± 9.5	46.6 ± 13.4	43.7 ± 10.1	< 0.001^a,b,c,d,e,f^
Non-HDL-C (mg/dl)	116.4 ± 23.4	172.1 ± 19.2	143.4 ± 13.1	197.0 ± 34.6	< 0.001^a,b,c,d,e,f^
Triglyceride (mg/dl)	132.0 ± 81.6	256.1 ± 118.3	127.8 ± 60.4	184.5 ± 96.5	< 0.001^a,,c,d,e,f^
Fasting glucose (mg/dl)	121.1 ± 50.4	119.4 ± 40.4	107.4 ± 20.9	108.5 ± 30.9	0.003^b,c^
Mean Gensini score	24.7 ± 38.1	28.2 ± 36.4	18.7 ± 28.1	26.5 ± 40.1	0.635
Mean SYNTAX score	7.1 ± 11.2	6.7 ± 11.3	5.4 ± 9.3	7.4 ± 11.6	0.733
Gensini score = 0 (%)	24.9	30.2	23.3	23.9	0.837
SYNTAX score = 0 (%)	55.1	60.5	58.1	54.3	0.821
Gensini score ≥ 20 (%)	34.7	27.9	30.2	34.2	0.799
SYNTAX score > 22 (%)	13.5	9.3	11.6	12.8	0.891

Data are expressed as percentage for categorical variables; chi-square test was used. Data are expressed as mean±standard deviation for continuous variables; one-way ANOVA was used; Statistically significant p-values are in bold. LSD test was performed for binary comparisons between groups and the p-value was set at 0.05. Significant differences were found between a) group I vs group II, b) group I vs group III, c) group I vs group IV, d) group II vs group III, e) group II vs group IV, f) group III vs group IV. LDL-C: low-density lipoprotein cholesterol; HDL-C: high-density lipoprotein cholesterol; Non-HDL-C: non-high-density lipoprotein cholesterol; BMI: Body Mass Index.

The variables age, BMI, smoking history, and percentage of patients with hypertension were not different between groups. The percentages of patients with diabetes mellitus and of patients receiving statin treatment were significantly different between groups (p = 0.004 and p < 0.001, respectively). Patients in Group 2 (LDL-C < median and non-HDL-C ≥ median) had the highest prevalence of diabetes mellitus and the lowest percentage of current statin treatment. The percentage of patients on statin treatment was the highest in Group 1 (LDL-C < median and non-HDL-C < median). Gender was significantly different from group to group (p = 0.036). Group 1 had the lowest percentage of females (LDL-C < median and non-HDL-C < median), while Group 4 had the highest (LDL-C ≥median and non-HDL-C ≥ median). Total cholesterol and LDL-C were present in high proportions in the groups with LDL-C ≥median and non-HDL-C ≥ median, but triglycerides was the highest in the group with LDL-C < median and non-HDL-C ≥ median (p < 0.001, p < 0.001 and p < 0.001, respectively).

The percentage of patients with Gensini or SYNTAX score of zero did not differ between groups (p = 0.837 and p = 0.821, respectively). Mean Gensini and SYNTAX scores, percentage of patients with Gensini score ≥ 20 and SYNTAX score > 22 were also not different between groups (p = 0.635, p = 0.733, p = 0.799 and p = 0.891, respectively). There was also no statistically significant correlation between LDL-C and Gensini or SYNTAX scores in any of the 4 subgroups. Additionally, no correlation was found between non-HDL-C and Gensini or SYNTAX scores in subgroups (Table 3).

**Table 3:** Correlation of LDL-C, non-HDL-C, Gensini and SYNTAX scores with Spearman's rho and p-value

	LDL-C < median Non-HDL-C < median n = 245 (group 1)		LDL-C < median Non-HDL-C ≥ median n = 43 (group 2)		LDL-C ≥ median Non-HDL-C < median n = 43 (group 3)		LDL-C ≥ median Non-HDL-C ≥ median n = 243 (group 4)	
	Gensini score	SYNTAX score	Gensini score	SYNTAX score	Gensini score	SYNTAX score	Gensini score	SYNTAX score
LDL-C	r = 0.118	r = 0.101	r = 0.088	r = 0.18	r = 0.127	r = 0.029	r = 0.031	r = 0.002
	p = 0.064	p = 0.115	p = 0.577	p = 0.910	p = 0.418	p = 0.853	p = 0.635	p = 0.972
Non-HDL-C	r = 0.046	r = 0.031	r = 0.190	r = 0.165	r = 0.104	r = 0.183	r = 0.025	r = 0.034
	p = 0.469	p = 0.624	p = 0.221	p = 0.290	p = 0.506	p = 0.240	p = 0.694	p = 0.596

LDL-C: low-density lipoprotein cholesterol; HDL-C: high-density lipoprotein cholesterol; Non-HDL-C: non-high-density lipoprotein cholesterol.

## Discussion

In the present study, we assessed the cross-sectional association between CAD severity/complexity and discordance between LDL-C and non-HDL-C numbers. While discordance was present between LDL-C and non-HDL-C in patients submitted to coronary angiography (15% of the sample), there was no difference regarding CAD severity and complexity between discordant and concordant groups.

Non-HDL-C represents the cholesterol content of all circulating atherogenic lipoproteins and it is not influenced by fasting conditions. Several studies have indicated that non-HDL-C is a better predictor of cardiovascular risk and mortality than LDL-C.^4^^,^^5^^,^^12^^,^^13^ It has been also reported that non-HDL-C was more closely associated with cardiovascular events than LDL-C in patients receiving statin therapy.^3^^,^^14^ There are some explanations for these states. Firstly, non-HDL-C includes VLDL and LDL cholestrols, and VLDL is also atherogenic.^15^^,^^16^ Secondly, non-HDL-C is an indirect measure of LDL-particles (LDL-p), and LDL-related atherosclerotic risk is better determined by the LDL-p number.^17^^-^^19^ Finally, non-HDL-C is correlated with the Apolipoprotein B (ApoB).^20^ ApoB carrying lipoproteins initiate and maintain the atherosclerotic process by entering and trapping within the arterial wall, so the total number of ApoB particles is a critical determinant of cardiovascular risk.^5^^,^^21^^-^^23^ To calculate non-HDL-C, no additional measurement beyond the routine lipid parameters is required, so no additional expense is made, which is an advantage for non-HDL-C over apoB.

LDL-p may be cholesterol-depleted or enriched. This variation causes discordance between LDL-C and non-HDL-C. The discordance rate in our study is similar to that of previous studies. In a study with 27,533 participants, prevalance of discordance was 11,6% and, in another study with 1,757 patients, it was 14.6 %.^7^^,^^8^ Also in a study conducted with aproximately 1.3 million adults, a similar discordance rate (15%) was found, especially at lower LDL levels.^24^ Discordance is high among subjects with high triglycerides level, lower HDL-C, dysglycemia and obesity.^7^^,^^25^^,^^26^

Coronary risk was found to be either underestimated or overestimated by LDL-C in individuals presenting discordance.^7^ Both LDL-C, non-HDL-C and discordance in relation to future cardiovascular events were evaluated in several studies. However, data about lipid parameters or discordance accurately predicting the severity or complexity of coronary atherosclerosis are limited and also contraversial.

In a study by Budde et al.,^27^ there was no relation between LDL-C and number, severity, and lenght of coronary lesions.^27^ Also, there was no relationship between LDL-C and coronary plaque volume, 3-vessel or left main coronary disease and severe coronary stenosis.^28^ In a study by Onat et al.,^29^ LDL-C was not a predictor of new-onset coronary heart disease.^29^ In two studies evaluating Gensini score and LDL-C relationship, LDL-C showed no significant difference when compared to high and low Gensini scores.^30^^,^^31^ In our study, LDL-C was not correlated to Gensini or SYNTAX scores. Non-HDL-C was found to be higher in patients with a Gensini score of 50 or greater than patients with a Gensini score of less than 50.^30^ There was a weak correlation (r = 0.113, p < 0.001) between non-HDL-C and Gensini score in a study by Zhang et al.^8^ In our study, the proportion of patients with high SYNTAX scores and high Gensini scores was low. Lack of association between CAD severity and non-HDL-C may have resulted from the relatively limited number of patients with severe CAD in our population.

There is a limited number of studies that evaluate the effect of discordance of LDL-C and non-HDL-C on coronary atherosclerosis severity. It was found that Gensini score was overestimated among patients with LDL-C greater than or equal to the median and non-HDL-C below the median.^8^ Shiiba et al.^32^ assessed the relationship between discordance and the mid-term outcome of coronary stent implantation. It was found that 3-vessel disease or left main tract disease did not differ among discordant and concordant groups, and discordance between LDL-C and non-HDL-C levels did not predict major adverse cardiovascular events after stent implantation.^32^ We assessed CAD severity by Gensini score and complexity by SYNTAX score, and these did not differ between discordant and concordant groups in our study.

### Study limitations

This study has several limitations. It has, for example, a retrospective design, which paves the way for the possibility of bias from unmeasured cofounders. One third of patients were using statins and the lack of association between discordance and CAD severity may have stemed from it. In addition, information about doses, species and duration of statin treatment were lacking. There is no absolute definition and standard cut-off values for the discordance of LDL-C and non-HDL-C. We used median values for our study population. Therefore, further large-scale prospective studies would be required to validate our results.

## Conclusion

While discordance was present between LDL-C and non-HDL-C (15% of patients), there is no difference regarding CAD severity and complexity between discordant and concordant groups. But the patients with LDL-C < median and non-HDL-C ≥ median present some high-risk features such as diabetes mellitus and higher triglyceride levels, and they may need further evaluation and close follow-up.
